# Fabrication of Annealed Gold Nanostructures on Pre-Treated Glow-Discharge Cleaned Glasses and Their Used for Localized Surface Plasmon Resonance (LSPR) and Surface Enhanced Raman Spectroscopy (SERS) Detection of Adsorbed (Bio)molecules

**DOI:** 10.3390/s17020236

**Published:** 2017-01-26

**Authors:** Rodica Elena Ionescu, Ece Neslihan Aybeke, Eric Bourillot, Yvon Lacroute, Eric Lesniewska, Pierre-Michel Adam, Jean-Louis Bijeon

**Affiliations:** 1 Laboratory of Nanotechnology, Instrumentation and Optics, UMR-CNRS 6281, Institute Charles Delaunay, University of Champagne, University of Technology of Troyes, 12 Rue Marie Curie CS 42060, 10004 Troyes CEDEX, France; pierre_michel.adam@utt.fr (P.-M.A.); jean-louis.bijeon@utt.fr (J.-L.B.); 2 Laboratory Interdisciplinaire Carnot de Bourgogne (ICB), UMR-CNRS 6303, University of Bourgogne Franche-Comte, 9 Avenue Alain Savary, 21078 Dijon CEDEX, France; ece.aybeke@dijon.inra.fr (E.N.A.); eric.bourillot@u-bourgogne.fr (E.B.); yvon.lacroute@u-bourgogne.fr (Y.L.); eric.lesniewska@u-bourgogne.fr (E.L.)

**Keywords:** annealed gold nanostructures, (bio)functionalization, improved LSPR and SERS sensitivity, human cytochrome b5, *trans*-1,2-bis(4-pyridyl)ethylene

## Abstract

Metallic nanoparticles are considered as active supports in the development of specific chemical or biological biosensors. Well-organized nanoparticles can be prepared either through expensive (e.g., electron beam lithography) or inexpensive (e.g., thermal synthesis) approaches where different shapes of nanoparticles are easily obtained over large solid surfaces. Herein, the authors propose a low-cost thermal synthesis of active plasmonic nanostructures on thin gold layers modified glass supports after 1 h holding on a hot plate (~350 °C). The resulted annealed nanoparticles proved a good reproducibility of localized surface plasmon resonance (LSPR) and surface enhanced Raman spectroscopy (SERS) optical responses and where used for the detection of low concentrations of two model (bio)chemical molecules, namely the human cytochrome b5 (Cyt-b5) and *trans*-1,2-bis(4-pyridyl)ethylene (BPE).

## 1. Introduction

Biosensors studie in the field of light-matter interactions require the development of active and sensitive metallic nanostructured substrates due to their ability to exhibit, in response to a light wave excitation, a localized surface plasmon resonance (LSPR) characterized by a collective oscillation of electrons in the metal and by a strong enhancement of local electromagnetic fields in the vicinity of nanoparticles [1,2,3,4]. Beside the physical investigations [5], the metallic nanoparticles (NPs) have been used in the development of either sensitive chemical [6,7,8] and biological LSPR [9,10,11,12,13,14] or surface enhanced Raman spectroscopy (SERS) [15,16,17,18,19] nanosensors. It has also been reported on the coupling between nanoparticles when strong SERS-exaltations of electromagnetic fields were noticed [20,21,22,23,24].

Very often active substrates rely mainly on noble metal colloidal clusters or nano-shells when their position, orientation, shape and optical properties are randomly distributed on a selected solid substrate. Thus, the size, shape and nature of metal nanoparticles contribute to a particular LSPR resonance response. It is well-known that the localized surface plasmons are radiative and can be coupled directly with the light due to the surface roughness (it does not require extra accessories such as prisms). Moreover, the strong confinement of the electromagnetic field in the vicinity of the nanoparticles and the plasmons propagate along the surface with an exponential decay from the surface at nanometric distances [3]. On the other hand, the surrounding medium is one of the key parameter in the determination of the position of the LSPR plasmon resonance peak position. Furthermore, the crystalline metallic nano-objects are expected to provide an ultra-high electromagnetic enhancement factor.

**Figure 1:**
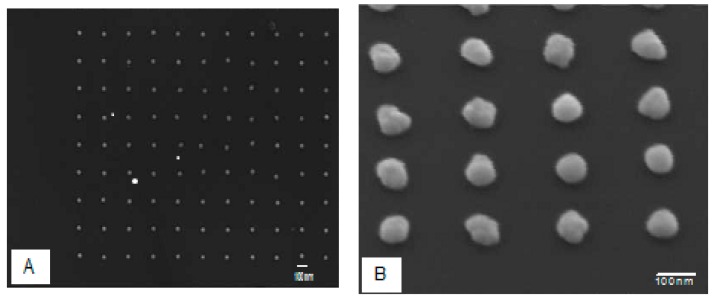
SEM images of nanoparticles obtained with (**A**) electron beam lithography (EBL) method. The nanoparticles are perfectly aligned within a well-defined array (**B**) and are not perfectly spherical or ellipsoidal.

The elaboration of nanoparticles requires the implementation of more or less complicated technologies such as the nanosphere lithography (NSL) [25] or the electron beam lithography (EBL). In the latter case, the nanoparticles are perfectly aligned and display a well-defined form (Figure 1A), while the EBL technique is limited to a working zone of a few hundred square microns and by the separation distances between nanoparticles reaching a lower limit, due to the size of the electron beam. However, if it is possible to obtain very close nanoparticles (few nanometers), the reproducibility and the production of such samples are very limited. Another major drawback of this technique, commonly observed (Figure 1B) is that the geometric shape of the nanoparticles is not perfectly spherical or ellipsoidal. Therefore, such nanometric defects can lead to an attenuation of the plasmon resonance and a broadening of the resonance peak. Interestingly, in the construction of nano-biosensors, the interaction between biological molecules and nanoparticles is not uniform onto the entire surface of nanoparticles and induce the appearance of signals commonly called “hot spots” which can produce higher plasmonic signals than those NPs of perfect geometries.

The aim of the present work is the development of active plasmonic substrate tailored with stable and randomly disposed annealed gold nanoparticles for biological and environmental applications. The main advantages of proposed substrates consist on the protocol simplicity, reproducibility and formation of nanoparticles of controllable particle sizes over large scaled surfaces in function of initially evaporated gold thin layers on glass. For the prove-of-concept, the LSPR and SERS optical investigations in the presence of aqueous solutions of cytochrome b5 (Cyt b5) and *trans*-1,2-bis(4-pyridyl)ethylene (BPE) are described and compared. It should be noted that SERS detection of cytochrome on silver nanoparticles has been reported by different groups [26,27,28].

## 2. Materials and Methods

### 2.1. Chemicals and Biomolecules

The optical performances of gold annealed substrates were evaluated through LSPR extinction and SERS measurements for two model molecules: one chemical compound (*trans*-1,2-bis-(4-pyridyl)ethylene (BPE)) and one hemoprotein (cytochrome b5). BPE is a standard symmetric test molecule for SERS experiments with a well-known assigned spectrum [29]. The experiments were performed with BPE at 10^−5^ M. On other hand, cytochrome b5 is an ubiquitous hemoprotein (16.7 kDa) found in microsomes of animals, plants, fungi and purple phototrophic bacteria [30]. Cyt b5 is involves in the reductive, oxidative and elongase enzymatic reactions as an obligate component or modifier [31]. It was reported that cytochrome b5 is required for full activity of flavonoid 3′, 5′-hydroxylase, a cytochrome P450 involved in the formation of blue flower colors [32].

In the present work, a modified human Cyt-b5 (10^−12^ M) was used for optical investigations. The 3D structure of Cyt-b5 is surrounded by unique cysteine, i.e., unique sulfhydryl residue. If Cyt-b5 is reduced by a reducing agent it is able to react directly with gold substrates via chemisorption. In this study, the Cyt-b5 was reduced by dithiothreitol (DTT) (20/1 moles ration) over 10 min. DTT was purchased from Sigma-Aldrich (Saint-Quentin Fallavier, France).

### 2.2. Preparation of Gold Nanostructures on Glass Substrates

Glass substrates were modified with annealed gold nanoparticles, i.e., controlled size, geometry and separation distance, using a homemade protocol. The soda lime glass substrates were cleaned with detergent to degrease the slide followed by extensively rinsing with deionized water. A surface pre-treatment that involves glow discharge cleaning at pressure of 2.8 × 10^−2^ Torr was applied on the cleaned substrates during one hour. Dimensions of used glass slides were 76 mm× 26 mm with a thickness of 1.1 mm. A total of 10 slides were placed together on the plate of the evaporator (MEB 300, Plassys Besteks, Marolles-en Hurepoix, France). The chamber was evacuated to a pressure lower than 2 × 10^−7^ Torr, and the selected gold film thickness was evaporated onto the slides from a resistively heated tungsten boat. Three different substrates were fabricated by evaporating the gold film at 3 nm, 5 nm and 12 nm of thicknesses.

**Figure 2:**
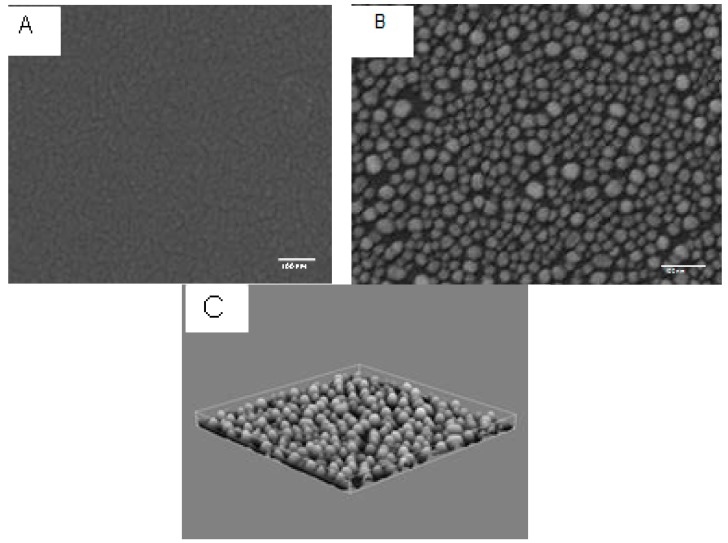
SEM images of a gold film (**A**) evaporated on glass obtained by physical vapor deposition method and (**B**) after annealing at 350 °C, for 1 h, in uncontrolled atmospheric conditions; (**C**) 3D AFM image (5 µm × 5 µm × 40 nm) of SEM image (B).

The plate was rotated during the evaporation process (deposition rate of 0.05 nm/s) to achieve homogeneous film deposition onto the glass slides. Finally, all the modified substrates were annealed at 350 °C for 1 h on a hot plate in atmospheric air uncontrolled (ambient conditions) when formation of various gold nanoparticles agglomeration was noticed. The impact of thermal annealing on a gold modified substrate is presented in Figure 2.

### 2.3. Instruments for Sample Characterization: SEM, AFM, XRD, SERS and LSPR Homemade Set-Up

SEM: The morphology of glasses unmodified and modified with different gold films before and after thermal annealing process was systematically analyzed by scanning electron beam microscopy (SEM 6500, Jeol Ltd., Tokyo, Japan). Figure 2A,B shows the morphology of evaporated and annealed gold films, respectively.

AFM: A 3D atomic force microscopy (AFM) image of annealed nanoparticles (Figure 2C), clearly shows the homogenate distribution of NPs in terms of sizes and distances interparticles.

XRD: The crystalline nature of nanostructures was investigated by X-ray diffraction (data not shown here) and demonstrated the face centered cubic crystal structure of the gold.

SERS: The Raman spectrometer is a HORIBA Jobin Yvon LabRam instrument (Horiba Ltd., Kyoto, Japan) equipped with a laser operating at 633 nm and power of 11 mW in a back reflection mode. The data were acquired with a ×50 or ×20 objective (with a numerical aperture N.A. = 0.8).

**Figure 3:**
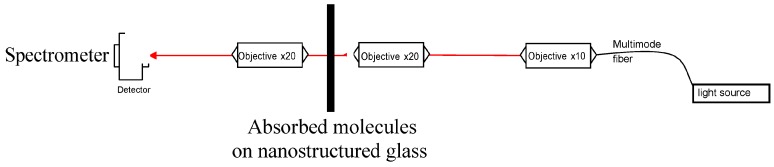
Optical set-up for LSPR extinction measurements.

LSPR homemade set-up: The optical analyses of the samples were performed by a home-built confocal transmission UV-Vis-NIR optical setup which allows acquiring the extinction spectra at different positions on the sample. The extinction spectrum is given by the absorbance formula—log (I//I_0_) where I_0_ is the intensity of the reference beam. The optical set-up is shown in Figure 3. Thus, optical fibers and lens were aligned; while the incident beam with spatial diameter of 30 µm was focused perpendicularly to the (bio)chemical modified nanostructured glass samples.

## 3. Results and Discussion

### 3.1. LSPR Characterization and SEM Images of Annealed Nanostructured

**Figure 4:**
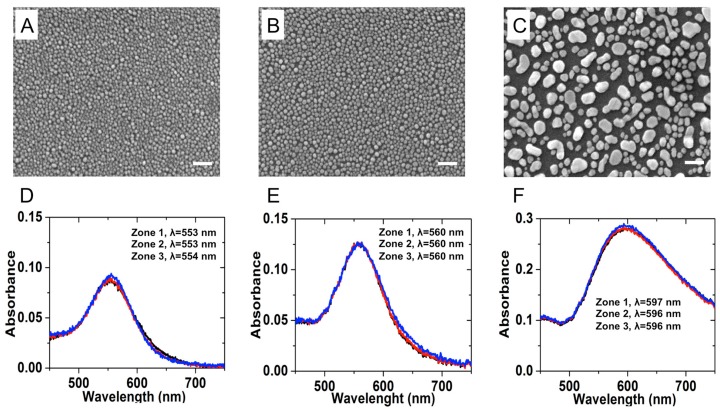
Scanning electron microscope (SEM) images of different gold nanoparticle substrates. The initial gold film thickness was (**A**) 3 nm for sample 1; (**B**) 5 nm for sample 2 and (**C**) 12 nm for sample 3. Scale bar is 100 nm. Extinction spectra of (**D**) sample 1; (**E**) sample 2 and (**F**) sample 3 are presented below corresponding SEM images. Red, blue and black lines present spectra collected from three different zones on each sample. The morphological and optical properties of each sample are listed in Table 1.

Following GDC, the samples were exposed to different gold evaporation times in order to vary the gold film thicknesses. Further, the annealing process after the metal deposition modified the morphology of the substrates. This process improved the homogeneous coverage of annealed nanoparticles on glass substrate. Figure 4 presents the comparative SEM images of three different substrates obtained by varying the initial gold film thickness. The gold film was evaporated at 3 nm, 5 nm and 12 nm of thickness for the sample 1, sample 2 and sample 3, respectively (Figure 4A–C). We investigated the physical and optical properties of active plasmonic substrates in the function of the initial gold film thickness. The LSPR wavelength was measured from three different zones on sample surface separated a few millimeters one from each other. The LSPR spectra of sample 1, sample 2 and sample 3 are presented in Figure 4D–F, respectively. The particle size distribution and the distances between annealed nanoparticles were calculated using ImageJ free software, developed by National Institutes of Health. The initial gold film thickness, the LSPR wavelength, the mean diameter and the interparticle distance of covered nanoparticles are listed in Table 1 for three different gold nanoparticle substrates.

### 3.2. LSPR Extinction Measurements for BPE and Cyt-b5

After the morphology investigation of three independent annealed substrates, the sample 2 was chosen for optical detection of selected chemical and biomolecule. Thus, the LSPR results using untreated-gold nanostructured samples show a remarkably stability of the position of the plasmon peak resonance (Table 1), confirming the prove-of-concept of a good homogeneity of the particle sizes and inter-particle gaps on all tested areas (sample 2).

**Figure 5:**
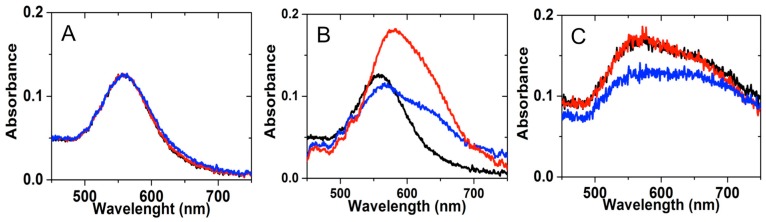
The extinction spectra of (**A**) Au nanoparticle substrate (sample 2); (**B**) dried BPE molecules and (**C**) dried Cyt-b5 molecules onto annealed Au nanostructures using 2 µL drop solution. Red, blue and black lines present spectra collected from three different zones on each sample.

In contrary, for treated-gold nanostructured samples with: droplets (2 µL) of BPE and Cyt-b5, respectively at different areas of the sample 2, following by the drying step for 1 h under a chemical hood, the resulted maximum intensity of plasmonic peaks and the full width at half maximum (FWHM) shows a variance for different particles zones since the absorbed molecules onto nanoparticles are not homogenous on the whole surface after drying the droplets of tested (bio)analytes (Cyt-b5 and BPE). These plasmonic behaviors can be attributed to the molecular interaction between the analyte and nanoparticles that modified the refractive index and shift the peak position in the extinction spectrum. In other words, the change in the position of the peak and in the FWHM is related with the quantity of adsorbed species on the analyzed nanostructured zone. Thus, Figure 5 presents the extinction spectra of (A) Au nanoparticle substrate (sample n° 2), (B) dried BPE molecules and (C) dried Cyt-b5 molecules onto annealed Au nanostructures using 2 µL drop solution.

**Figure 6:**
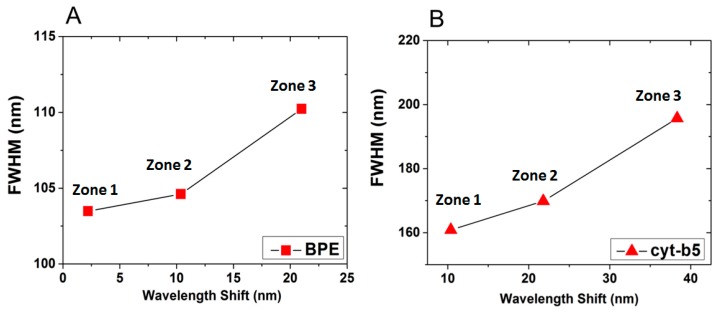
Relation between FWHM and wavelength shift for (**A**) BPE and (**B**) Cyt-b5 molecules using the recorded spectra from three independent zones exposed to drops of specific biomolecules solution namely BPE and Cyt-b5, respectively.

Table 2 summarizes the LSPR results reported in Figure 5, while Figure 6 presents the relation between FWHM (Figure 6A) and wavelength shift (Figure 6B) that are proportional for both tested molecules (BPE and Cyt-b5). Consistently, the LSPR results proved the ability of homemade annealed gold nanostructured substrates to detect different type of (bio)molecules such as BPE and Cyt-b5, respectively.

### 3.3. SERS Measurements for BPE and Cyt-b5

**Figure 7:**
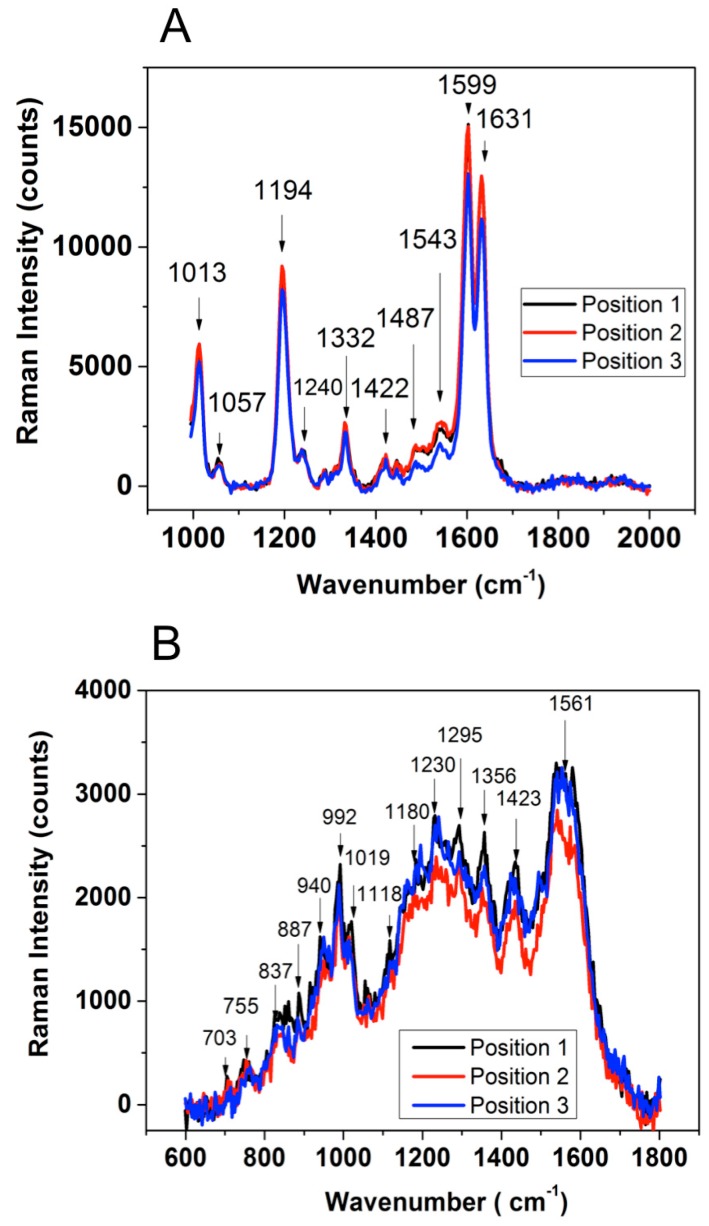
SERS spectra of adsorbed BPE (**A**) and Cyt-B5 (**B**) molecules onto sample 2.

SERS spectra were acquired from fresh deposited aqueous droplets of (bio)molecules (BPE and Cyt-b5, respectively). Thus, the treated-nanostructured substrates were investigated for their homogeneity on the same glass-sample and between different prepared glass-samples. Moreover, three different zones separated on each sample by few millimeters were also SERS investigated. Figure 7A presents the SERS spectra of adsorbed BPE molecules on gold nanostructured sample n° 2. It was found that the fresh prepared aqueous solutions of BPE molecules have non influence on the stability of the spectrum versus the position of the analyzed zone. The molecular signatures of BPE are well compared with the published results [29] and summarized in Table 3.

In the case of reduced Cyt-b5 biomolecule in aqueous solution, its SERS spectrum shows the presence of two specific bands at 1338 cm^−1^ and 1303 cm^−1^ (Figure 7B). These SERS results are in excellent agreement with the published Raman [33] spectrum proving the high efficiency of authors proposed metallic substrates. Further, the comparison of Raman spectra for Cys-b5 is given in Table 4.

Interestingly, the resulted SERS spectra of Cyt-b5 is quite different from that reported by Kakita et al. due to a different selected excitation wavelength: 633 nm instead of 532 nm.

## 4. Conclusions

Low concentrations of BPE and human Cyt-b5 molecules were detected optically using LSPR/SERS spectroscopies and annealed gold films for 1 h at 350 °C using pre-treated glow- discharge cleaned glasses. These substrates were carefully characterized in function of different parameters: initial metal thickness deposition, resulted sizes and distance between neighboring nanoparticles. It was found that SERS spectra were highly sensitive when tested molecules were adsorbed, whereas logical resonant LSPR plasmonic shifts were observed. It is expected that such active nanostructured surfaces to be used in the development of water pollutants plasmonic nano-biosensors.

## Figures and Tables

**Table 1:** Statistical information of annealed gold nanoparticles about their morphology and LSPR position over three independent prepared three glass nanostructured samples.

Morphology	Sample 1: (A)	Sample 2: (B)	Sample 3: (C)
Initial gold film thickness (nm)	3	5	12
Mean diameter of nanoparticles (nm)	5–8	8–10	50–80
LSPR wavelength (nm)	553	560	596

**Table 2:** FWHM and wavelength LSPR maximum shift for AuNPs substrate, with adsorbed BPE and Cyt-b5 for the three selected zones.

	AuNPs (Sample 2)			
	FWHM		*λmax* (nm)	
	75.992		558.1	
	BPE		Cytochrome b5	
Zone	FWHM	*λmax* (nm)	FWHM	*λmax* (nm)
**1**	110.232	579.896	169.84	579.896
**2**	104.608	568.475	160.85	568.475
**3**	103.483	560.383	195.722	596.42

**Table 3:** SERS vibrational spectra of BPE molecules reported in 1996 [29] and those obtained on the present study using annealed gold nanostructures developed on pre-treated glow-discharge cleaned glasses.

Symmetry	Calculated Frequencies (cm^−1^) [21]	Published SERS Spectra of BPE (cm^−1^)	Present SERS Spectra of BPE (cm^−1^)
13	986	1008	1013
12	1071	1064	1057
11	1097		
10	1145	1200	
9	1201	1200	1194
8	1224	1244	1240
7	1340	1314	1292
6	1363	1338	1332
5	1409	1421	1422
4	1498	1493	1487
3	1560	1544	1543
2	1609	1604	1599
1	1682	1640	1631

**Table 4:** SERS vibrational spectra of Cyt-b5 with estimated peak value from published work [33] and compared with the spectra obtained on the present study using annealed gold nanostructures developed on pre-treated glow-discharge cleaned glasses.

Published SERS Spectra of Cyt-b (cm^−1^)	Present SERS Spectra of Cyt-b5 (cm^−1^)
1580	1561
1400	1423
1338	1356
1303	1295
1250	1230
1180	1180
1120	1118
1080	1019
1050–900 (5 peaks)	1019–992–940–887
800–900	837
780	775
670	703
